# Venetoclax Combined with Intensive Chemotherapy: A New Hope for Refractory and/or Relapsed Acute Myeloid Leukemia?

**DOI:** 10.3390/jcm13020549

**Published:** 2024-01-18

**Authors:** Ramy Rahmé, Thorsten Braun

**Affiliations:** 1 Hematology Department, Avicenne Hospital, Assistance Publique-Hôpitaux de Paris, 93000 Bobigny, France; 2 Faculty of Medicine, Université Sorbonne Paris Nord, 93017 Bobigny, France; ramy.rahme@aphp.fr

**Keywords:** AML, leukemia, refractory, relapsed, chemotherapy, venetoclax

## Abstract

**Background**. Primary resistance of acute myeloid leukemia (AML) to the conventional 3 + 7 intensive chemotherapy and relapses after first-line chemotherapy are two highly challenging clinical scenarios. In these cases, when allogeneic stem cell transplantation is feasible, patients are usually retreated with other chemotherapeutic regimens, as transplantation is still considered, nowadays, the only curative option. **Methods**. We discuss the mechanisms behind resistance to chemotherapy and offer a comprehensive review on current treatments of refractory/relapsed AML with a focus on novel approaches incorporating the BCL-2 inhibitor venetoclax. **Results**. Alas, complete remission rates after salvage chemotherapy remain relatively low, between 30 and 60% at best. More recently, the BCL-2 inhibitor venetoclax was combined either with hypomethylating agents or chemotherapy in refractory/relapsed patients. In particular, its combination with chemotherapy offered promising results by achieving higher rates of remission and bridging a substantial number of patients to transplantation. **Conclusions**. Venetoclax-based approaches might become, in the near future, the new standard of care for refractory/relapsed AML.

## 1. Introduction

Based on response and survival rates after intensive chemotherapy (IC), the ELN (European LeukemiaNet) risk classification (the latest update of which was in 2022) distinguishes three prognostic subgroups of acute myeloid leukemia (AML) at diagnosis: favorable, intermediate and adverse [1]. This classification can guide the choice of first-line treatment. By IC, we mean the traditional ‘3 + 7’ cytotoxic regimen combining an anthracycline (daunorubicin or idarubicin administered for 3 days) with cytarabine (a nucleoside analogue administered for 7 days). This combination has remarkably stood the test of time for 40 years [2]. In patients aged less than 70 years, this long-established IC embodies the backbone of treatment in favorable- and intermediate-risk AMLs and, to some extent, in some adverse-risk cases. Moreover, allogeneic transplantation is indicated in first complete remission (CR) in intermediate- and adverse-risk AMLs, but not in favorable cases if molecular CR is achieved [3,4]. Still, across the three subgroups, 20% of patients do not obtain CR after upfront IC [5,6], and 40 to 50% experience a relapse without transplantation [7].

As is the custom, primary refractory diseases and relapses can be treated with intensive approaches if, in general, patients are still considered eligible for allogeneic stem cell transplantation (SCT). Indeed, SCT is still considered today the only reliable option with curative potential for these patients when CR is achieved. A systematic review analyzing 24 studies in refractory/relapsed (R/R) AMLs found a median CR rate of 30% (range 3.3–75%) after salvage chemotherapy, and patients who managed to receive SCT had better survival compared to those who did not [8].

In this paper, we first discuss the mechanisms behind resistance and relapse after IC. In actual fact, little is known about these mechanisms and some clues are currently emerging from molecular studies. Second, the contemporary strategies used to treat R/R AML are exposed. Finally, we give some insight on therapeutic approaches incorporating the BCL-2 inhibitor venetoclax as these novel strategies seem highly efficacious in inducing remission and bridging patients to SCT.

## 2. How Does Intensive Chemotherapy Work in AML, and Why Do AMLs Resist Chemotherapy?

Even though a substantial number of AML patients will achieve CR after IC, the majority of patients will eventually relapse through complex and mostly unexplainable mechanisms. A prevailing idea is that relapse emerges from a small resistant population to chemotherapy—the so-called leukemia stem cells (LSCs)—that is characterized by a self-renewal potential [9]. Yet, an analysis of AML cells straightly surviving chemotherapy in mice and patients, i.e., the remaining cells that persist after chemotherapy was administered, did not reveal an enrichment of LSCs, suggesting that additional mechanisms are involved in relapse [10,11]. Furthermore, although the selection of clones harboring resistance mutations following IC (e.g., TP53) is rarely observed in relapsed AMLs, LSCs at relapse are inherently more resistant to chemotherapy than their original counterparts at diagnosis [12,13].

**Figure 1:**
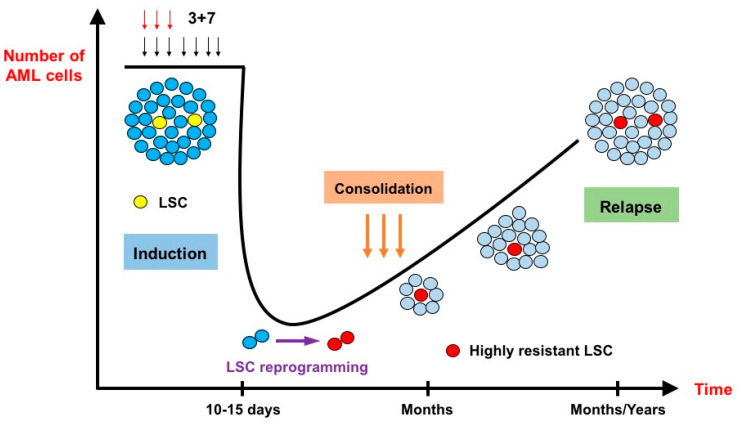
The effects of intensive chemotherapy on bulk acute myeloid leukemia cells and leukemia stem cells (inspired by Duy et al. [17]). After administration of the conventional 3 + 7 intensive chemotherapy, very few leukemic cells survive. At nadir, 10 to 15 days after the start of chemotherapy, the lingering cells do not necessarily belong to the leukemia stem cell (LSC) population. They are blocked in a senescence–like state and undergo an LSC reprogramming, rendering them more resistant to chemotherapy than their original counterparts. Relapse originates from these highly resistant novel LSCs.

The genotoxic stress induced by IC can lead to different types of response in the leukemic cells such as apoptosis, senescence or cell cycle arrest [14,15]. These responses are essentially TP53-dependent and closely related to the extent of DNA damage induced by IC, as senescence, for instance, is the result of sublethal stress [16]. In a recent elegant work conducted by Duy et al. [17], the residual fraction of cells that persist after IC, i.e., cells harvested in patients who did not recover yet from hematological toxicity following chemotherapy, was assessed for gene signature. While LSC gene signatures were not enriched at nadir, a senescence-like phenotype was identified in these lingering cells. Interestingly, upon recovery, these same cells manifested an enrichment of LSC gene signatures. The authors hypothesized that a LSC programming should occur during this senescence-like resilient phenotype that follows chemotherapy and would explain how newly derived stress-adaptive LSCs are enriched at relapse (Figure 1). This process seems to play a critical role in relapse and could potentially be targeted, as ATR (i.e., ataxia telangiectasia and Rad3-related protein: a kinase involved in sensing DNA damage and activating the DNA damage checkpoint) was identified as a major player in the induction of this cellular state [18,19,20]. Furthermore, BCL-2 inhibition can also target the senescence state in multiple models of cellular aging [21,22].

LSCs share many characteristics with normal hematopoietic stem cells [23]: they divide slowly and self-renew, making conventional cytotoxic anti-proliferative therapies less effective on them compared to highly proliferative myeloblasts. Metabolic reprogramming in LSCs transcends the conventional Warburg effect as many metabolic pathways are involved in their maintenance: PI3K/AKT and mTOR [24,25], pentose phosphate pathway [26], glutaminolysis [26], oxidative phosphorylation (OXPHOS) [26] and fatty acid oxidation [27]. As a matter of fact, LSCs rely on OXPHOS for ATP generation instead of glycolysis and lactic acid fermentation; this metabolic state results in a relative vulnerability to the production of ROS in mitochondria via electron transport, which can force LSCs out of quiescence and induce apoptosis. In response to this threat, LSCs regulate ROS levels by inducing autophagy and upregulating the expression of the hypoxic response transcription factor HIF-1α, even in normoxic conditions [28,29]. Indeed, the residual AML cells that persist after chemotherapy have exhibited gene signatures consistent with a high OXPHOS status [10]. Cytotoxic drugs used in AML treatments are known to stimulate ROS production [30,31]. Thus, targeting this redox balance can be critical to induce cell death in LSCs [32]. On another level, lower mitochondrial permeabilization (i.e., priming) in LSCs has been associated with resistance to chemotherapy [33]. Overall, an improvement in understanding the effects of IC on LSCs and AML cells should hopefully lead to changes in the design of upfront and salvage regimens in order to reduce the risk of primary resistance/relapse and to enhance CR rates after relapse.

## 3. How Are Relapsed/Refractory AMLs Treated Nowadays?

Primary resistance to IC and relapses remain among the most challenging scenarios in the management of AML. One should distinguish between early relapse, meaning relapse within 6 months after first CR, and late relapse (>6 months) as a response to salvage therapy, and the overall survival rates are significantly different [1,34,35]. Treatment decisions are historically based on this distinction. For instance, in patients aged ≥ 50 years, intensive salvage still offers a longer post-relapse survival than low-dose chemotherapy and the best supportive care when the duration of first CR is longer than 12 months [36]. In addition, Breems et al. identified the following prognostic factors for survival after relapse (i.e., the European Prognostic Index (EPI)): length of the relapse-free interval after first CR (cut-off 18 months), cytogenetics at diagnosis, age at relapse (cut-off 35 years) and history of prior SCT [37]. Finally, the GOELAMS index integrates the following factors to predict survival after relapse: duration of first CR (cut-off 1 year), *FLT3-ITD* positive status and high-risk cytogenetics [38].

Intensive regimens for R/R AMLs were not compared in clinical trials (Table 1). Early studies in the 1980s support the use of high-dose cytarabine (HiDAC) as a salvage regimen [39,40]. HiDAC remains the most used regimen but is associated with unsatisfactory results with a CR rate of 30% [41]. Notably, in the large randomized French BIG-1 study (on behalf of the Filo, ALFA and SFGM-TC study groups), refractory patients who failed induction IC received either high-dose (HDAC, i.e., 3 g/m^2^) or intermediate-dose (IDAC, i.e., 1.5 g/m^2^) cytarabine as a salvage regimen. The CR rate was higher in HDAC vs. IDAC (51.6% vs. 39.5%), although this difference was not statistically significant (*p* 0.081) (Abstract, Blood (2023) 142 (Supplement 1): 967) [42].

The addition of other drugs to HiDAC such as etoposide [41] and, more recently, the conjugated monoclonal anti-CD33 antibody gemtuzumab ozogamicin [43] failed to improve CR rates and survival. In the 1990s, other regimens combining several drugs such as MEC (mitoxantrone, etoposide, intermediate-dose cytarabine) [44], EMA (etoposide, mitoxantrone, continuous cytarabine) [45], FLAG-IDA (fludarabine, cytarabine, G-CSF, idarubicin) [46] were associated with higher CR rates, up to 60–65 [43,47,48,49]. Still, the median duration of second CR was somehow disappointing, ranging from 5 to 10 months. More recently, randomized trials showed that targeted therapies perform better than intensive chemotherapies in R/R cases (Table 2). For instance, the FLT3 inhibitor gilteritinib induced higher CR rates (21.1 vs. 10.5%) and longer survival (9.3 vs. 5.6 months) than salvage chemotherapy in R/R FLT3-mutated AMLs. Yet, these responses were very short-lived in both arms (2.8 vs. 0.7 months) [50]. Likewise, R/R patients receiving quizartinib, another FLT3-ITD inhibitor, fared better than those receiving chemotherapy (CR rates 48 vs. 27%); yet, CR durations were still highly insufficient (survival 6.2 vs. 4.7 months) [51]. On another level, IDH inhibitors offered promising results in R/R cases. For the IDH1 inhibitor ivosidenib, the CR rate was 21.6% and the median duration of response was 8.2 months [52]. For the IDH2 inhibitor enasidenib, those rates were in the same range, respectively, 19.3% and 5.8 months [53].

**Table 1:** Intensive chemotherapy regimens used in the treatment of refractory/relapsed acute myeloid leukemia.

Regimen	CR Rate	Early Death	Median Post-CR2 Remission	References
HiDAC HiDAC + Etoposide	31% (*n* = 67) 38% (*n* = 66)	NA	11.9 months 25 months	Vogler et al., Leukemia (1994) [41]
MEC	66% (*n* = 32)	6% (2/32)	9 months	Amadori et al., JCO (1991) [44]
EMA	65% (*n* = 96)	5% (5/96)	5.5 months	Thomas et al., Leukemia (1999) [45]
FLAG-IDA	63% (*n* = 19)	0%	7–10 months	Parker et al., Br J Haematol (1997) [46]
CLAG-M	30% (*n* = 60)	5%	12 months	Halpern et al., Haematologica (2019) [47]
Clofarabine/Cytarabine	46% (*n* = 46)	8.7%	9 months	Becker et al., Br J Haematol (2011) [48]
GO-HiDAC	32% (*n* = 37)	VOD = 0%	8.9 months	Stone et al., Leuk Res (2011) [43]
GO-FLA (Pediatric)	83% (*n* = 29)	VOD = 21%	14 months	Dhunputh et al., Br J Haematol (2022) [49]

CR: complete remission; CR2: second complete remission; HiDAC: high-dose cytarabine; MEC: mitoxantrone, etoposide, intermediate-dose cytarabine; EMA: etoposide, mitoxantrone, continuous cytarabine; FLAG-IDA: fludarabine, cytarabine, G-CSF and idarubicin; CLAG-M: cladribine, cytarabine, G-CSF and mitoxantrone; GO: gemtuzumab ozogamicin; FLA: fludarabine and cytarabine.

## 4. BCL-2 Inhibition in the Frontline Treatment of AML

The B-cell lymphoma-2 (BCL-2) family of proteins includes multiple regulators of apoptosis. Each member plays a specific role in the intrinsic apoptotic pathway through the fine regulation of mitochondrial outer membrane permeabilization (MOMP). All BCL-2 family proteins share the BH3 domain (i.e., BCL-2 homology 3) that is responsible for direct physical interactions between them on the mitochondrial outer membrane [54]. Based on their functions, three groups are individualized: (1) anti-apoptotic proteins or suppressors (BCL-2, BCL-X L, BCL-W, MCL-1, BFL-1/A1); (2) pro-apoptotic effectors as pore formers (BAX, BAK, BOK); and (3) pro-apoptotic BH3-only proteins that are subdivided into activators (BID, BIM, PUMA) and sensitizers (BAD, NOXA) [55,56]. Suppressors inhibit the activity of pro-apoptotic members, thus preventing the following events: the formation of pores in the mitochondrial outer membrane, the resulting release of cytochrome C into the cytoplasm and its interaction with pro-caspase 9 and Apaf-1 to form the apoptosome, triggering downstream cleavage and the activation of caspase 3 that ultimately orchestrates apoptotic cell death [57].

**Figure 2:**
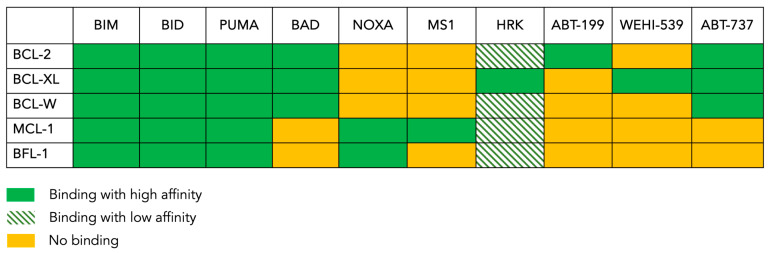
The BH3 binding map. This figure shows the pattern of interaction between the anti-apoptotic BCL-2 family members present in the tested cells (rows) and the pro-apoptotic peptides or drugs (columns) used in the BH3 profiling assay. BIM, BAD and PUMA are pan-sensitizers that inhibit all the inhibitors. ABT-199 (venetoclax) inhibits only BCL-2, whereas ABT-737 inhibits BCL-2, BCL-XL and BCL-W. MS1 specifically inhibits MCL-1, while NOXA inhibits BFL-1 in addition to inhibiting MCL-1. WEHI-539 only inhibits BCL-XL, and HRK is mainly a BCL-XL inhibitor but can also inhibit other anti-apoptotic proteins with lower affinities.

The anti-apoptotic protein BCL-2 was found to be overexpressed in many hematological malignancies, in particular, chronic lymphocytic leukemia (CLL). Unlike CLL, BCL-2 is not universally overexpressed in AML [58,59,60]. Nonetheless, its mere level of expression does not necessarily imply dependence, as other suppressors can still potentially contribute to leukemogenesis; this is also true the other way round [56]. The dependence on BCL-2 family pro-survival members can be assessed via a technique called “BH3 profiling”. This test is based on our understanding of how the different members of BCL-2 family interact with each other. A summary of these specific and complex interactions is shown in Figure 2. In addition, the binding affinities of BH3-only proteins to the five anti-apoptotic members are also known. Consequently, by exposing mitochondria to known concentrations of BH3 peptides and measuring the resulting MOMP, the dependence for survival on specific anti-apoptotic proteins can be determined [57,61,62]. For instance, the activators BIM, BID and PUMA bind to all anti-apoptotic proteins; they are considered “pan-sensitizers”. The remainder of the peptides have more selective binding patterns. For example, HRK only binds to BCL-XL. Thus, a response from the HRK peptide will indicate a dependence on BCL-XL. Similarly, NOXA only binds to MCL-1, and a response in this setting will indicate a dependence on MCL-1. In parallel, BAD for example can bind to three different proteins, and therefore, it cannot discriminate between the three. Nevertheless, a response from a BAD peptide would indicate that at least one of its binding partners in involved in MOMP. In addition to BH3 peptides, the use of pharmacological agents such as ABT-199 (venetoclax) and WEHI-539, which bind to BCL2 and BCL-XL, respectively, can refine the profiling. While this technique originally relied on the isolation of mitochondria as heavy membrane preparations, modern techniques use permeabilized cells; this allows for an MOMP measurement to take place via flow cytometry and microscopy in addition to bulk measurements using a fluorescent plate reader or cytochrome C Western blots. Many AML cell lines and primary AML blasts have a dependence on BCL-2 based on this assay [63,64], an observation that has paved the way toward using BCL-2 inhibition as a therapeutic strategy for AML patients [56,65].

**Figure 3:**
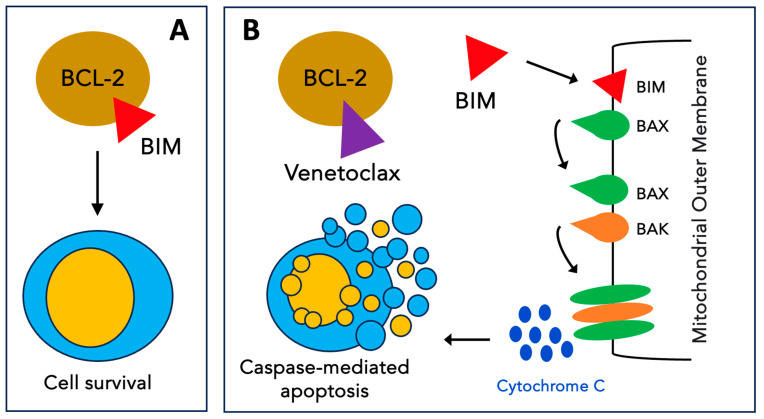
Mechanism of action of venetoclax. (**A**) The antiapoptotic protein BCL-2 inhibits apoptosis by sequestering pro-apoptotic BH3-only proteins such as BIM. (**B**) Venetoclax selectively binds to BCL-2, freeing BH3-only activators that trigger BAX and BAK oligomerization on the mitochondrial outer membrane. The formation of these pores results in the release of cytochrome C into the cytoplasm triggering events that lead to apoptosis.

Venetoclax (VEN), formerly known as ABT-199, is a BH3 mimetic that targets BCL-2 with high selectivity [66] (Figure 3). In the first preclinical study on AML [67], single-agent VEN showed high efficacy, both in vitro (at nanomolar concentrations) and in vivo, in BCL2-dependent cell lines. The following preclinical works focused on combination strategies to enhance VEN activity and overcome resistance. For instance, in one study, VEN synergized with chemotherapeutic agents (daunorubicin or cytarabine) to induce the apoptosis of patient-derived AML cells [68]. Notably, while VEN monotherapy increased MCL-1 protein levels, the combination reduced these levels, resulting in this synergistic effect on cell death. Furthermore, synergy has been observed with hypomethylating agents [69,70,71]. Other combinations have included alvocidib (CDK9 inhibitor) [72], pevonedistat (Nedd8 inhibitor) [73], GDC-0980 (PI3K/mTOR inhibitor) [74], idasanutlin (Mdm2 inhibitor) [75,76], and cobimetinib (MEK inhibitor) [77], and these have led to similar findings, mainly via the direct or indirect reduction in MCL-1 levels. Finally, direct MCL-1 inhibitors were combined with VEN (A-1210477 [78], VU661013 [79] and AMG 176 [80]) and uniformly enhanced cell death.

The first clinical trial of VEN in AML patients was a phase two single-agent study in R/R cases. The overall response rate was 19% with a median duration of response of only 48 days [81]. The most common grade 3 or higher adverse events were febrile neutropenia (31%), hypokalemia (22%) and pneumonia (19%). Unlike reports in CLL, no tumor lysis syndrome occurred in this AML cohort. Thereafter, clinical development focused on the frontline treatment setting. Given the synergistic activity observed in preclinical studies, VEN was combined with either a hypomethylating agent (azacitidine or decitabine) or low-dose cytarabine (LDAC). Moreover, hypomethylating agents and LDAC were chosen as backbone therapies because they were considered, at the time, to be the standard of care for newly diagnosed (ND) elderly AML patients unfit for IC. In an open-label dose-escalation trial [82], previously untreated elderly people who were ineligible for IC were treated with VEN and azacitidine (AZA) or decitabine (DEC). Three different target doses were chosen for VEN: 400, 800 and 1200 mg. Accordingly, six distinct groups of patients were formed. While in no cohort was the maximal tolerated dose (MTD) reached, the 1200 mg dose was associated with a high frequency of gastrointestinal adverse effects (nausea 82%, diarrhea 64%). Accordingly, the 400 and 800 mg cohorts with both hypomethylating agents were expanded. In the intention-to-treat population (*n* = 145), the CR rate was 37% and CRi was 30%. After a median follow-up of 15 months, the median overall survival was 17.5 months. In the 400 mg arm, CR/CRi rates were 71% and 74% for AZA and DEC, respectively. The most common grade 3 or higher adverse events were thrombocytopenia (47%) and febrile neutropenia (42%) [82,83]. Another study investigated LDAC plus VEN in previously untreated patients who were aged over 60 years and ineligible for IC [84]. A daily VEN dose of 600 mg was chosen as the expansion phase dose. Of the 82 patients who received 600 mg, the CR and CRi rates were 26% and 28%, respectively. More specifically, 33 patients had had prior exposure to hypomethylating agents: the CR/CRi rate in this group was low at 33%, whereas this rate was higher in patients who were treatment-naive, at 62%. The median survival was 10.1 months, and the median duration of response was 8.1 months. The most common grade 3 or higher adverse events were febrile neutropenia (42%) and thrombocytopenia (38%).

The randomized trial VIALE-A (NCT02993523) enrolled untreated patients with AML who were ineligible for standard IC, because of preexisting comorbidities or older age (≥75 years), to receive AZA plus either VEN (target dose 400 mg) or placebo [85]. The primary end-point of the study was overall survival. The intention-to-treat analysis included 431 patients (288 AZA/VEN and 145 AZA/placebo) with a median age of 76 years in both groups. At a median follow-up of 20.5 months, the median survival was 14.7 months in AZA/VEN and 9.6 months in AZA/placebo (*p* < 0.001). Notably, both CR and CR/CRi rates were significantly higher in AZA/VEN: 36.7% vs. 17.9% (*p* < 0.001) and 66.4% vs. 28.3% (*p* < 0.001), respectively. Febrile neutropenia episodes were more frequent in AZA/VEN (42% vs. 19%). Based on these results, the combination of the BCL-2 inhibitor VEN with the hypomethylating agent AZA is currently approved for newly diagnosed AML patients aged ≥75 years and for younger patients who are not eligible for IC.

The combination AZA/VEN appears to be active across the cytogenetic and genomic AML subgroups. Importantly, most patients with high-risk features have overall high response rates compared to AZA alone, making this combination an appealing therapy for patients who have a predicted low likelihood of responding to conventional IC. Lower response rates were specifically observed in some subgroups such as in patients who were previously treated with a hypomethylating agent and those who harbored a TP53 mutation [83,84]. All in all, up to 40% of patients do not obtain CR/CRi after AZA/VEN treatment. Many studies have investigated the mechanisms of resistance in these refractory patients and revealed some clues: the monocytic differentiation of AML blasts which is associated with resistance [86,87,88,89]; the loss of BAX expression in AML cells rendering them unable to undergo apoptosis [90]; dependence on CLBP, a chaperonin involved in maintaining mitochondrial intermembrane integrity [91]; and mutations in the FLT3 or PTPN11 genes [92], causing a higher expression of other members of the BCL-2 family including BCL-XL and MCL-1 [93,94]. In contrast, AML cases with either IDH1 or IDH2 mutation have a higher sensitivity to venetoclax compared to unmutated cases [95,96]. More recently, Waclawiczek et al. developed and validated a flow cytometry-based assay that measured the protein expression of BCL2, BCL-XL and MCL1 in leukemic stem cells [97]. A score was then calculated (MAC score for “Mediators of apoptosis combinatorial score”); this scoring predicted the initial response to AZA/VEN with a positive predictive value of 97%. Finally, Bhatt et al. showed, both in patient-derived xenografts and human primary samples, that resistance to BH3 mimetics (i.e., VEN as an BCL2 inhibitor and S63845 as an MCL-1 inhibitor) was characterized by decreased mitochondrial priming as measured via BH3 profiling; this was due to alterations in BCL-2 family proteins that vary among cases but not due to acquired mutations in leukemia driver genes [98]. Importantly, decreased/limited mitochondrial priming can lead to “minority MOMP” and limited caspase activation that is insufficient to trigger apoptosis. Conversely, this caspase activity leads to DNA damage that, in turn, promotes genome instability, cellular transformation and more resistance to treatments [99,100,101].

Based on additional BH3 profiling data, it was shown that AML blasts primed to apoptosis are highly sensitive to cytotoxic agents [33,102]. Therefore, VEN was also combined with various intensive chemotherapy regimens in the frontline treatment of AML. A phase I trial showed that VEN can be safely combined with 7 + 3 induction at a daily dose of 200 mg for 10 days in ND AML cases with patients aged <60 years (Abstract, Blood (2019) 134 (Supplement_1): 3908) [103]. Another phase 1b study (CAVEAT study) used an attenuated induction regimen of cytarabine and idarubicin (5 + 2) in patients aged >65 years [104], and the MTD of VEN was not reached with a daily dose of 600 mg. The CR and CRi rates were 41% and 31%, respectively. Specifically, the CR rate was 68% in de novo AMLs compared to 9% in secondary AMLs, and a poor survival was reported for TP53-mutated cases (3.6 months). VEN doses were reduced in consolidation courses (cytarabine 2 days, idarubicin 1 day and VEN 14 days) due to hematotoxicity. Moreover, the FLAG-IDA regimen combined with VEN induced CR/CRi in 85% of ND cases [105]. Furthermore, VEN was combined with the CLIA regimen (cladribine, idarubicin and high-dose cytarabine) as a frontline treatment for younger patients <65 years [106]. The study included 50 patients with a median age of 48 years: 84% obtained CR and 82% of the responding patients achieved minimal residual disease (MRD) negativity. The estimated 12 month survival was 85%. Finally, VEN was combined with a lower intensity chemotherapy regimen as an induction course (i.e., CLAD-LDAC for cladribine plus low-dose cytarabine) followed by a consolidation phase that alternated courses of AZA/VEN with courses of CLAD-LDAC/VEN (NCT03586609) in ND AML patients [107]. The updated results were presented as a poster at the ASH 2023 meeting (Abstract, Blood (2023) 142 (Supplement 1): 4256) [108]. In this cohort of 123 patients, the CR and CR/CRi rates were 74% and 85%, respectively. MRD negativity was achieved in 78% of responders. Of note, 93.5% of patients achieved their best response after one cycle, and 39% were bridged to transplant which offered a survival benefit (no SCT: median OS 49.8 months vs. SCT: median OS not reached). All in all, these results suggest that the combination of VEN with intermediate intensity chemotherapy regimens might improve outcomes in comparison to AZA/VEN. Before moving these regimens to frontline therapy, future studies with head-to-head comparisons seem mandatory.

## 5. Venetoclax-Based Approaches for Refractory or Relapsed AML

More recently, a small number of studies assessed VEN-based treatments in R/R cases. First, VEN was combined with epigenetic modulators. When combined with AZA in two studies, CR rates ranged from 44 to 59% [109,110]. One study found 1 and 2 year overall survival rates of 49.6 and 39.0%, respectively [109]. In another study, treatment with AZA/VEN (*n* = 25) yielded similar outcomes compared to standard chemotherapy (*n* = 38) [110]: CR/CRi 36% vs. 37%; median survival 287 vs. 285 days. More specifically, prior exposure to AZA was associated with lower CR/CRi (13% vs. 41%). Furthermore, the combination of VEN with the oral hypomethylating agent ASTX727 (decitabine + cytidine deaminase inhibitor cedazuridine) achieved significant efficacy in R/R AMLs with an overall response rate of 53% [111]. Recently, novel AZA/VEN-based combinations were reported. The triple combination of VEN, AZA and the histone deacetylase inhibitor tucidinostat in a phase II study yielded a high overall response rate of 75% [112]. Moreover, compared to AZA/VEN in R/R patients, the triplet VEN, AZA and homoharringtonine offered significantly higher rates of CR/CRi (63.8 vs. 40.9%), overall survival (22.1 vs. 16.0 months) and event-free survival (14.3 vs. 2.3 months) with a median follow-up of 14.7 months [113,114]. The promising results of these triplets in R/R AML are worth further exploration in larger studies.

Moreover, the results of VEN-based chemotherapies in an R/R setting show deeper remissions than previous regimens do (Table 3). DiNardo et al. reported the results of a phase IB/II study of VEN (target dose 400 mg) combined with FLAG-IDA in R/R AML (*n* = 39). The overall response rate was 70–75% with 69% of patients achieving a measurable residual-disease composite CR. Fifty-six percent of patients proceeded to SCT, and the 1-year survival rate was 78% after transplant [105,115]. In addition, VEN was combined with cladribine and low-dose cytarabine (CAV). The overall response rate was 90.5% with an estimated 1-year OS and EFS of 91.7% and 74.9%, respectively [116]. Similarly, VEN combined with HAM (HiDAC and mitoxantrone) led to a CR/CRi of 92% and 62.5% of evaluable patients who achieved negative MRD [117]. In our experience (manuscript under preparation), the FLAG-IDA-VEN regimen is indeed associated with high CR rates and can bridge a significant number of patients to SCT with a clear survival benefit after a median follow-up of more than one year. As a matter of fact, we observed a CR rate of 86% after the induction course with no early death, and ultimately, 59% proceeded to SCT. After a median follow-up of 410 days, 59% of patients were alive (Abstract, Blood (2023) 142 (Supplement 1): 1520) [118]. Lastly, VEN was combined with CPX-351 (i.e., a liposomal fixed-ratio formulation of cytarabine and daunorubicin) in a phase Ib/II study (NCT03629171) that enrolled 33 patients. This combination yielded lower complete responses (CR 15%, CRi 24%) and a high rate of early death (4- and 8-week mortality: 9 and 21%, respectively). Responses were more prevalent in patients not previously exposed to VEN (57% vs. 37%). All in all, 60% were able to receive SCT. After a median follow-up time of 20.7 months, the median overall survival was 6.4 months with SCT, allowing extended survival (Abstract, Blood (2023) 142 (Supplement 1): 4259) [119].

## 6. Conclusions

In less than a decade, BCL-2 inhibition has become an essential therapeutic strategy in AML. Although the use of VEN as monotherapy has yielded disappointing results, its combination with a hypomethylating agent, and more recently, with intensive chemotherapy, has led to high response rates and durable remissions, even in the unfavorable-risk group. Nowadays, resistance to BCL-2 inhibition in AML is better understood and should lead to a better selection of patients who would benefit the most from BCL-2 inhibitors. In parallel, novel combinations targeting other BCL-2 family members, such as BCL-XL and MCL-1, could improve responses and outcomes in the future. Moreover, mitochondrial priming upon BCL-2 inhibition can be further modulated using different targeting strategies aiming at enhancing cell death [98,120,121,122,123].

In patients with refractory AML and those who experience relapse after frontline intensive chemotherapy regimens, allogeneic stem cell transplantation remains, when feasible, the only curative option. Therefore, bridging therapies need to ensure high rates of remission and low toxicity. VEN-based therapies, in particular, the FLAG-IDA-VEN combination, seem to embody the best available approaches today in this setting. Progress should be made to considerably decrease primary refractoriness and relapses with novel therapeutic combinations based on a better understanding of how chemotherapy works in AML. Until then, we think that VEN-based chemotherapies offer, indeed, a new hope for R/R AML.

## Figures and Tables

**Table 2:** Targeted therapies used in the treatment of refractory/relapsed acute myeloid leukemia.

Targeted Therapy	CR Rate	Median Survival	References
Gilteritinib (*n* = 247) vs Chemo (*n* = 124)	21.1% 10.5%	9.3 months 5.6 months	Perl et al., NEJM (2019) [50]
Quizartinib (*n* = 245) vs Chemo (*n* = 122)	48% 27%	6.2 months 4.7 months	Cortes et al., Lancet Oncol (2019) [51]
Ivosidenib (*n* = 258)	21.6%	8–9 months	DiNardo et al., NEJM (2018) [52]
Enasidenib (*n* = 239)	19.3%	9.3 months	Stein et al., Blood (2017) [52]

**Table 3:** Venetoclax combined with intensive chemotherapy for the treatment of refractory/relapsed acute myeloid leukemia.

Regimen	N	Age Range	Response Rates	Median Follow-Up	Survival	References
FLAG-IDA-VEN	39	18–68	ORR 70%	15.8 months	Median OS 27 months 1-year OS 60%	DiNardo et al. (2021) [105] Desikan et al. (2022) [115]
Cladribine + AraC + VEN	21	16–68	ORR 90.5% CR/CRi 28.6%	3.8 months	Estimated 1-year OS 91.7% 1-year EFS 74.9%	Li et al. (2022) [116]
Mitoxantrone + HiDAC + VEN	12	40–70	CR/CRi 92%	N/A	N/A	Röllig et al. (2022) [117]

CR: complete remission; CRi: CR with incomplete count recovery; ORR: overall response rate; EFS: event-free survival; OS: overall survival; VEN: venetoclax; AraC: cytarabine; HiDAC: high-dose cytarabine; FLAG-IDA: fludarabine, cytarabine, G-CSF and idarubicin.

## Data Availability

Not applicable.
